# Unfilled Natural Rubber Compounds Containing Bio-Oil Cured with Different Curing Systems: A Comparative Study

**DOI:** 10.3390/polym14122479

**Published:** 2022-06-18

**Authors:** Chesidi Hayichelaeh, Phattarawadee Nun-Anan, Mili Purbaya, Kanoktip Boonkerd

**Affiliations:** 1 Department of Materials Science, Faculty of Science, Chulalongkorn University, 254 Phayathai Road, Bangkok 10330, Thailand; h.chesidi@gmail.com (C.H.); phattarawadee.anan@gmail.com (P.N.-A.); milipurbaya3107@gmail.com (M.P.); 2 Center of Excellence on Petrochemical and Materials Technology, Bangkok 10330, Thailand; 3 Green Materials for Industrial Application Research Unit, Faculty of Science, Chulalongkorn University, 254 Phayathai Road, Bangkok 10330, Thailand

**Keywords:** natural rubber, processing oil, bio-oil, aromatic oil, palm oil, soybean oil, curing system, sulfur, peroxide, cure kinetics

## Abstract

This study focuses on the properties of unfilled natural rubber compounds containing bio-oils cured with a peroxide curing system and then discusses the comparisons to those cured using the sulfur system from our previous work. Two types of bio-oils, i.e., palm oil and soybean oil, were used, and distillate aromatic extract (DAE)-based petroleum oil was employed as a reference. The bio-oils caused no significant change in the vulcanization of rubber compounds cured using peroxide. However, the compounds containing bio-oils and cured with sulfur showed a faster vulcanization than the ones with DAE. The bio-oils strongly affected the crosslink density of rubber compounds in both curing systems. The use of bio-oils caused a low crosslink density due to the possible implication of curing agents to bio-oil molecules. The properties of rubber compounds dependent on the different levels of crosslink density were also investigated. The results revealed that when the crosslink density increased, the modulus, tensile strength, and hardness of the rubber compounds increased and the elongation at break and compression set decreased. The use of bio-oils in the rubber compounds cured with different curing systems gave low modulus at 300% strain, tensile strength, and hardness but high elongation at break and compression set when compared to the ones with DAE. However, no significant change was observed for the compression set of the rubber compounds cured using sulfur. With the presence of bio-oils, the properties of rubber compounds cured with sulfur system deteriorated less than those of the ones cured with peroxide.

## 1. Introduction

Given that raw rubber does not retain its shape nor retract to its original form after a large deformation, it requires vulcanization to become useful. Vulcanization is a chemical reaction that occurs under high temperatures of 120 °C–200 °C to induce bonding among rubber chain segments and generate a 3D network structure (crosslinking point) within the rubber matrix. This process generates a rubber material with the desired shape and size. Two types of vulcanization or curing systems, i.e., sulfur and peroxide curing systems, are widely used in unsaturated rubber compounds. The crosslink types formed after vulcanization vary with the vulcanization system. The rubber compounds cured with sulfur contain long rubber chains that are covalently linked via sulfide bridges ranging from mono- and di- to poly-sulfidic bridges. As a curing agent, peroxide forms a radical on the rubber chains, and two rubber radicals bind to form the –C–C– crosslink. Compared with the sulfur crosslink, the–C–C–bond of the peroxide curing system is rigid and strong. Thus, the sulfur curing system shows flexibility. However, the thermal stability of –C–C– bond is higher than that of the sulfur crosslink, resulting in good heat resistance and a low compression set. In addition to the different crosslink types, the levels of crosslink density within the rubber matrix also affect the rubber properties, including hardness, tensile strength, elongation at break, tear strength, compression set, tension set, creep, fatigue resistance, and swelling resistance. The crosslink density slightly changes several properties, including abrasion resistance, gas permeability, glass transition temperature (low temperature flexibility), electrical conductivity, heat conductivity, and chemical stability [1].

Natural rubber (NR) is a green polymer obtained from *Hevea brasilliensis* and has a high molecular weight and a high amount of cis-1,4-polyisoprene, which can crystallize under stretching (strain-induced crystallization) and thus generate superior tensile strength and good resistance against crack growth [2,3,4,5,6]. Owing to the high molecular weight of NR, the preparation of the NR compound is divided into several processes, i.e., mixing, milling, and the extrusion of mixing. Processing oil is required in NR compounds because it can act as an internal lubricant and make polymers with high molecular weight acceptable for processing [7]. In addition, processing oil can improve the properties of rubber compounds, i.e., flex life and low temperature, enhance filler dispersion within the rubber matrix of filled rubber compounds, improve end-use properties, and magnify the volume of rubber compounds for cost reduction [8]. Petroleum-based processing oil has been used in rubber compounds for over 150 years, and distillate aromatic extract (DAE) is the main choice for unsaturated rubber due to their good compatibility. However, polycyclic aromatic hydrocarbons (PAHs), a DAE component, were identified as carcinogens—a toxic substance [9]—and, thus, have been banned by the European Union since 1 January 2010, especially in tire application [10,11]. Hence, safe processing oils with a low level of PAHs (lower than 3 wt%), i.e., treated distillate aromatic extract (TDAE) and mildly extracted solvate (MES), have been developed. The feasibility of using TDAE and MES in unfilled rubber compounds from NR, SBR, and their blend was studied by comparing the properties of rubber compounds containing DAE. The three different types of processing oil, i.e., DAE, TDAE, and MES, showed good compatibility with rubbers. In addition, different temperatures changed the compatibility, as confirmed by the oil uptake in the lightly crosslinked rubbers. DAE and TDAE showed better compatibility than MES [12]. The different types of processing oil had a minor effect on the Mooney viscosity, cure rate index, and tensile strength of the rubber compounds, except for the compounds from NR. In addition, the use of MES in NR compounds provided low cure rate index and tensile strength [13].

Bio-oils show potential as an eco-friendly, renewable, and sustainable resource replacement for DAE oil in rubber compounds. The bio-oils used in rubber compounds are mostly modified forms. Sahakaro and Beraheng (2011) investigated the effect of epoxidized palm oil (EPO) and epoxidized soybean oil (ESBO) on the cure and mechanical properties of carbon black-filled rubber compounds. They also compared the properties between these compounds and those with DAE. DAE exhibited a positive effect on vulcanization—shortest cure time and highest torque difference. ESBO induced worse vulcanization—longer cure time and lower torque difference—compared with EPO. Owing to the low torque difference (referred to as low crosslink density) of the rubber compounds with ESBO, the rubber compounds exhibited low mechanical properties (modulus at 100% and tensile strength) [14]. Boontawee and coworkers (2017) studied the effect of benzyl esters synthesized from bio-oils on the properties of carbon black-filled SBR compounds using compounds with aromatic oil as a reference. Benzyl esters promoted vulcanization—shorter scorch and cure times and a lower crosslink density—compared with aromatic oil. Owing to the low crosslink density caused by the oil molecules, the rubber compounds with benzyl esters showed low hardness, modulus, and tensile strength but high elongation at break and compression set [15]. However, the modification of bio-oil increases the cost and time. Therefore, the present work focused on the feasibility of using unmodified bio-oils as processing oil in rubber compounds.

The properties of unfilled natural rubber compounds containing bio-oils, i.e., palm oil (PO) and soybean oil (SBO), using sulfur curing system have been reported by authors who demonstrated that the bio-oils had a huge influence on the properties of natural rubber compounds, such as cure characteristics, crosslink density, and mechanical properties [16]. So, this research work aimed to study the properties of unfilled NR compounds containing bio-oils cured with peroxide systems and to comparatively discuss with those cured with sulfur system [16]. Two types of bio-oils, PO and SBO, were used, and DAE-based petroleum oil was selected as a reference. The properties related to the vulcanization of rubber compounds, such as scorch time, cure time, torque difference, cure reaction rate constant, activation energy, and crosslink density, were investigated. The mechanical properties, i.e., modulus at 300% strain, tensile strength, elongation at break, hardness, and compression set of NR compounds containing oils, were also reported. Furthermore, the properties of rubber compounds containing bio-oils and cured with different curing systems, i.e., sulfur and peroxide curing systems, were compared and discussed.

## 2. Materials and Methods

### 2.1. Materials

Standard Thai Rubber (STR 5L), locally produced in Thailand, was used in this study. Two types of bio-oils, i.e., PO and SBO, locally produced in Thailand were used. The viscosities of PO and SBO were 233 and 213 cP, respectively, as measured by a Brookfield KU-2 Viscometer [16]. Tudalen 65 (H&R ChemPharm, Bangkok, Thailand), with aromatic (45%), naphthenic (26%), and paraffinic (29%) components, was used as DAE oil. The rubber compounding ingredients were zinc oxide (ZnO) (Global Chemical, Samut Prakan, Thailand), stearic acid (Imperial Chemical, Bangkok, Thailand), and sulfur (Siam Chemicals, Bangkok, Thailand). Dibenzothiazyl disulfide (MBTS) was purchased from Flexys, Bruxelles, Belgium. Dicumyl peroxide (DCP) (A.F. Supercell Co., Ltd., Rayong, Thailand) was used as the peroxide curing agent.

### 2.2. Preparation of NR Compounds

**Figure 1:**
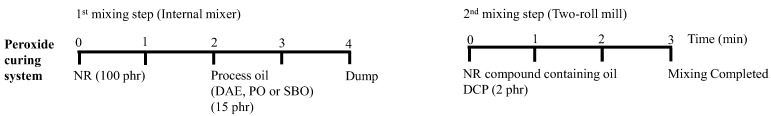
Mixing steps for the preparation of rubber compounds.

Sulfur-cured rubber compounds were prepared as previously described [16]. For peroxide-cured rubber compounds, unfilled NR compounds containing different types of processing oils, i.e., DAE, PO, and SBO, were prepared as shown in Table 1. A two-step mixing procedure was adopted for the preparation of rubber compounds (Figure 1). For the first step, the NR compounds containing oils were mixed using an internal mixer at the following conditions: fill factor of 0.8, initial temperature of 60 °C, and rotor speed of 55 rpm. NR was masticated for 2 min before the processing oils were added and mixed continuously for 2 min. The processing oils were added and mixed for 1 min. Afterward, the NR compounds were transferred to the internal mixer and sheeted by a two-roll mill. The NR compounds were kept for 24 h at room temperature before the curing agents were added. In the second step, the curing agents were mixed with the NR compounds using a two-roll mill. The NR compounds were then sheeted by a two-roll mill and kept for 24 h at room temperature before further characterizations.

### 2.3. Testing and Characterizations

#### 2.3.1. Swelling Test

Samples of NR compounds without processing oil cured by DCP at 170 °C were prepared and cut into sheets with a size of approximately 1 cm × 1 cm × 0.2 cm. The swelling of NR vulcanizates in different types of oils was tested. The samples were immersed in oil with a fixed volume of 30 mL for 30 days. The percentage of the swelling of the samples was calculated using Equation (1):

(1) $$Swelling\;\left(\%\right)=\left(\frac{w_{1}-w_{0}}{w_{0}}\right)\times 100$$

where *w*_0_ and *w*_1_ are the weights of a sample before and after swelling in oil, respectively.

#### 2.3.2. Cure Behavior

The cure characteristics of NR compounds were measured using a moving die rheometer in accordance with ASTM D5289 at various isothermal temperatures, i.e., 170 °C, 180 °C, 190 °C, and 200 °C. Scorch and cure times were calculated by using different percentages of cure, in which the scorch time was considered at 10% cure and cure time at 90% cure. The cure rate index, which is equal to [100/(cure time-scorch time)], was also calculated. For the testing of properties, i.e., crosslink density, mechanical properties, hardness, and compression set, all rubber compounds were vulcanized to their cure times by compression molding at 170 °C.

#### 2.3.3. Crosslink Density

The crosslink density of NR vulcanizates was investigated by the equilibrium swelling method. Samples prepared with a fixed size of 10 mm × 10 mm × 2 mm were swollen in 20 mL of toluene at a room temperature. After swelling for 7 days, the samples were dried at 105 °C for 24 h. The volume fraction of polymers in the swollen rubber (*V_r_*) was determined using Equation (2), and crosslink density (*X_c_*) was calculated using Equation (3) [17]:

(2) $$Vr=\left(m_{1}/\rho_{1}\right)/\left(\left(m_{1}/\rho_{1}\right)+\left(m_{2}/\rho_{2}\right)\right)$$

where *m*_1_ is the weight of polymer, *m*_2_ is the weight of solvent in the swollen NR vulcanizate at equilibrium swelling, *ρ*_1_ is the density of rubber vulcanizates before swelling, and *ρ*_2_ is the density of solvent.

(3) $$Xc=\left(-\left(\ln1-Vr\right)+Vr+XVr^{2}\right)/\left(V_{2}\left(Vr^{1/3}-Vr/2\right)\right)$$

where *X* is the polymer–solvent interaction parameter (0.391) and *V*_2_ is the molar volume of the solvent (106.3 cm^3^/mol).

#### 2.3.4. Tensile Properties

The tensile properties, i.e., modulus at 300% strain, tensile strength, and elongation at break, were tested using a universal testing machine (Tinius Olsen, Tinius Olsen TMC, Horsham, PA, USA). The rubber vulcanizates were cut to a dumbbell shape specimen by using a die cut type I of ISO37 standard. The specimens were tested at room temperature with an extension speed of 500 mm/min.

#### 2.3.5. Hardness

The hardness of rubber vulcanizates containing different types of processing oils and cured by sulfur and peroxide was tested using a durometer (Frank GmbH, Hamburg, Germany) in accordance with the ASTM D2240 test procedure, and the results are expressed in Shore A.

#### 2.3.6. Compression Set

The compression set of rubber compounds containing different types of processing oils was tested in accordance with ASTM D395. The rubber vulcanizates were compressed for 25% deformation and heated in a hot air oven (Memmert, ITS Co., Ltd., Bangkok, Thailand) at 100 °C for 22 h. After heating, the rubber vulcanizates were released from the compression to restore their original shape for 30 min. The compression set of the rubber vulcanizates was calculated as shown in Equation (4):

(4) $$Compression\;set\left(\%\right)=\left(\frac{t_{0}-t_{1}}{t_{0}-t_{s}}\right)\times 100$$

where *t*_0_ is the thickness of the samples; *t*_1_ is the thickness of the samples after testing; *t_s_* is the thickness of the spacer bar.

The properties of rubber compounds, such as swelling in oils, crosslink density, modulus, tensile strength, elongation at break, hardness, and compression set, were tested in three samples. The average value and standard deviation were calculated and reported.

## 3. Results and Discussion

### 3.1. Solubility Aspects

**Figure 2:**
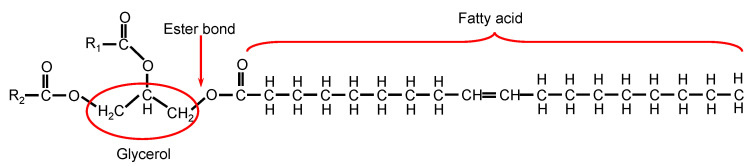
Postulated structure of bio-oil.

**Figure 3:**
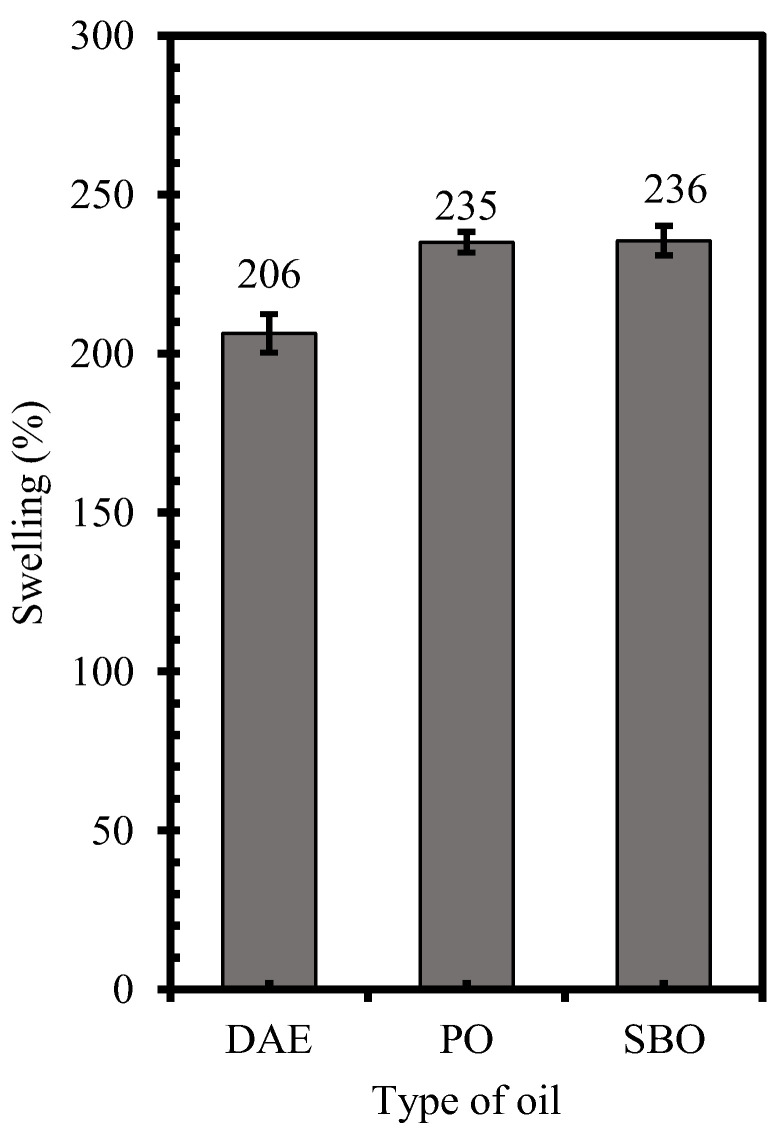
Percentage of swelling of NR in oil.

Given the different chemical structures of rubber and oil, the compatibility of these components depends on the solubility parameter (δ). A solubility parameter difference of oil and rubber close to zero, i.e., |δ_oil_ − δ_rubber_|→ 0, promotes their compatibility. The solubility parameter can be calculated from the volume of substance per mole and the molar attraction constant related to cohesive energy (E_coh_) using the equation described by Small [18,19]. The solubility parameters of NR and DAE oil were 16.9 and 17.5 J^1/2^/cm^3/2^, respectively [12], and the difference in their solubility parameter was 0.6 J^1/2^/cm^3/2^. The solubility parameters of bio-oils, i.e., PO and SBO, were reported. The solubility parameters of PO are 18.3 and 18.4 J^1/2^/cm^3/2^ [20,21,22], whereas those of SBO are 16.2 and 16.8 J^1/2^/cm^3/2^ [20,23,24]. The chemical structure of bio-oils, i.e., PO and SBO, comprises three fatty acids attached to a glycerol backbone via an ester linkage (Figure 2). Bio-oils also contain slight amounts of free fatty acids [25], which are also released by the hydrolysis of triglycerides [26]. The major fatty acids in PO are oleic-, palmitic-, lenolenic-, and stearic- acids, while as the major fatty acids in SBO are linoleic-, oleic-, palmitic-, lenolenic-, and stearic- acids [15,27,28,29,30]. The unsaturated/saturated ratio of fatty acids in PO and SBO is in the range of 1.04-1.32 and 5.35-6.14, respectively. Thus, the calculation of actual solubility parameter of bio-oil is complicated. The swelling of NR in the different types of oils was applied to investigate the compatibility of these components (Figure 3). The results revealed that both bio-oils, i.e., PO and SBO, showed higher percentage of swelling than DAE. Thus, these bio-oils promote the compatibility with NR molecule.

### 3.2. Cure Characteristics

**Figure 4:**
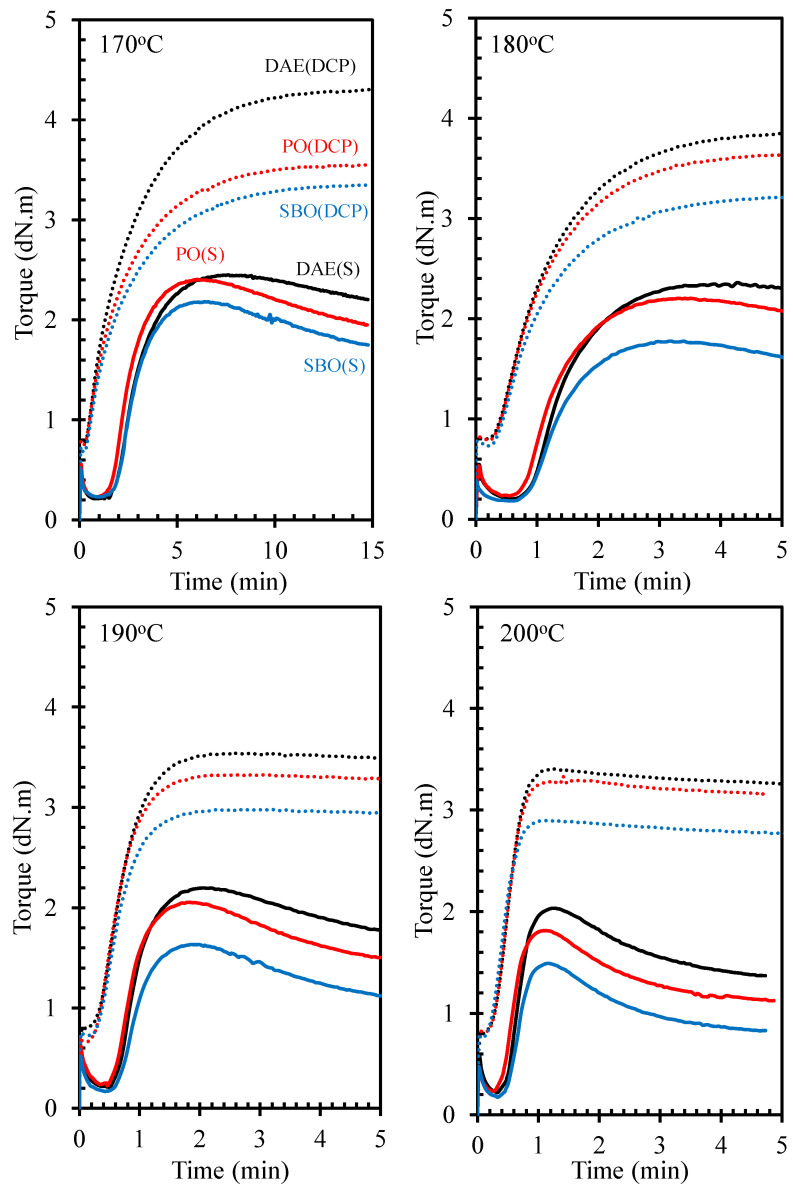
Torque–time curve of NR compounds containing different types of oils with various curing systems.

Figure 4 shows a typical torque-time curve of NR compounds containing processing oils with peroxide curing systems and compared with those using sulfur curing system from [16]. The curing system showed a strong influence on the cure curve behavior of the NR compounds throughout the curing period in this study. The results revealed that the cure curves of the peroxide-cured NR compounds containing processing oil showed a marching behavior, and those of the sulfur-cured NR compounds exhibited a reversion behavior. The behaviors of cure curves in the over curing period depend on the competition between the formation and breakage of the crosslinking points within the rubber matrix. Given the different thermal stabilities of crosslinking points, the thermal stability of C–C in the peroxide curing system was higher than that of sulfidic crosslinks. The bond dissociation energy of crosslinking points of rubber compounds was in the order of C–C (343.2 kJ/mol) > C–S (276.2 kJ/mol) > S–S (205.1 kJ/mol) [1]. Therefore, during the over curing period, the crosslinks of the sulfur-cured NR compounds decomposed more easily than those of the peroxide-cured NR compounds. Generally, this condition caused the sulfur-cured NR compounds to show a reversion behavior during the over curing period. On the other hand, the cure curves at high temperatures, especially at 200 °C, of the rubber compounds cured with peroxide showed reversion behavior. This is due to the degradation of NR molecules and the reduction of crosslinking points within the rubber matrix.

**Figure 5:**
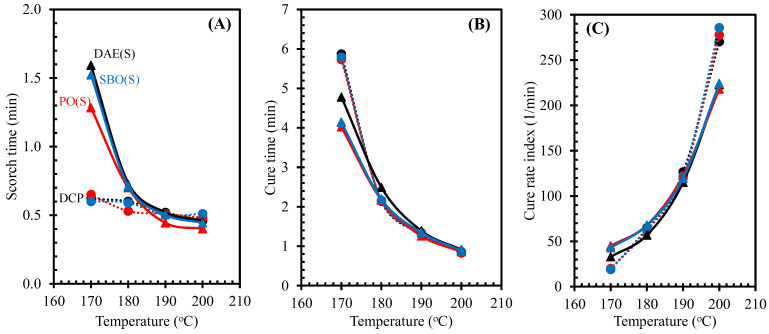
Scorch time (**A**), cure time (**B**), and cure rate index (**C**) of NR compounds containing different types of oils with various curing systems as a function of curing temperatures.

Figure 5A–C illustrate the cure characteristics, such as scorch time, cure time, and cure rate index, respectively, of NR compounds containing processing oils from different curing systems. Owing to the effect of different curing systems, the scorch time of NR compounds under peroxide curing was shorter than that under sulfur curing. This finding may be attributed to the different reactions of premature vulcanization. By contrast, the use of sulfur curing system led to a shorter cure time than the use of peroxide curing system due to the different behaviors during the over curing period, i.e., marching for DCP- and reversion for S-curing systems. However, the different curing systems gave similar level on the scorch time and cure time at high curing temperatures, i.e., 190 and 200 °C.

The processing oils did not significantly influence the scorch and cure times of the NR compounds using peroxide curing system, which was in contrast with the sulfur-cured NR compounds. The different types of processing oils strongly affected the cure time of sulfur-cured NR compounds, in which the NR compounds containing bio-oils, i.e., PO and SBO, had a shorter cure time compared with the one with DAE oil. For bio-oil compositions containing certain amounts of free fatty acids, these fatty acids can react with ZnO, enhancing the speed of vulcanization reaction. Thus, the sulfur-cured NR compounds containing both bio-oils, i.e., PO and SBO, showed a shorter cure time and higher cure rate index than the one containing DAE oil [16]. However, the similar levels on the cure time and cure rate index of the rubber compounds were observed when the high temperatures were applied.

### 3.3. Cure Kinetics

**Figure 6:**
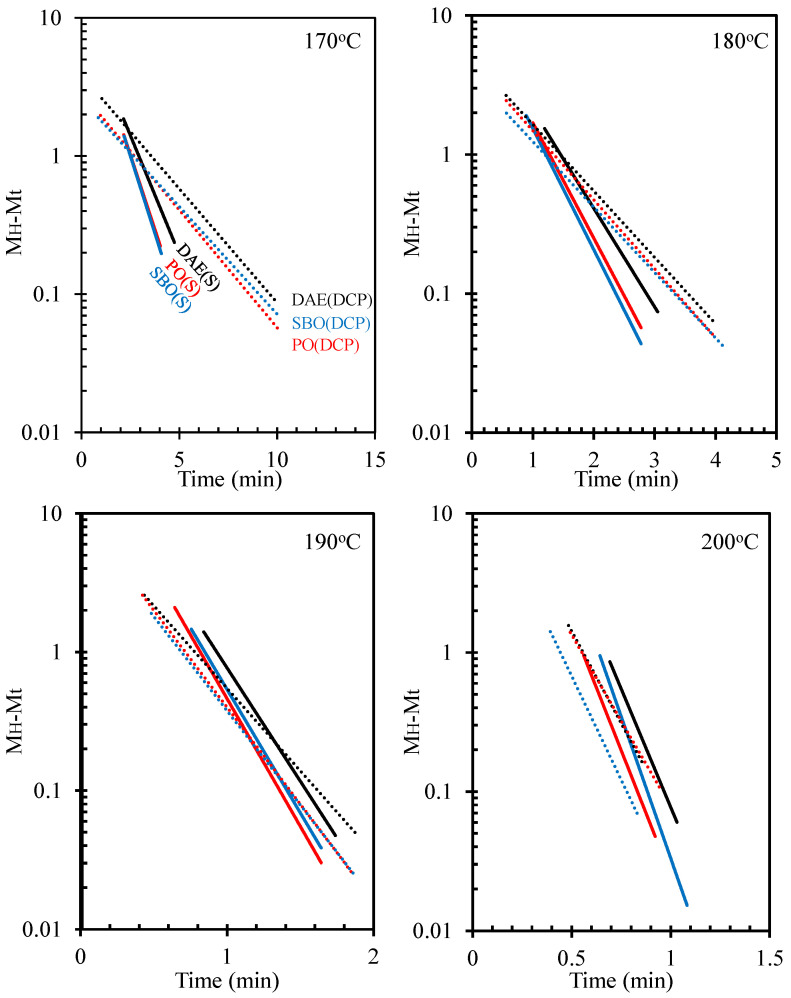
ln (M_H_ − M_t_) versus time (t) of NR compounds containing different types of oils under various curing systems.

**Figure 7:**
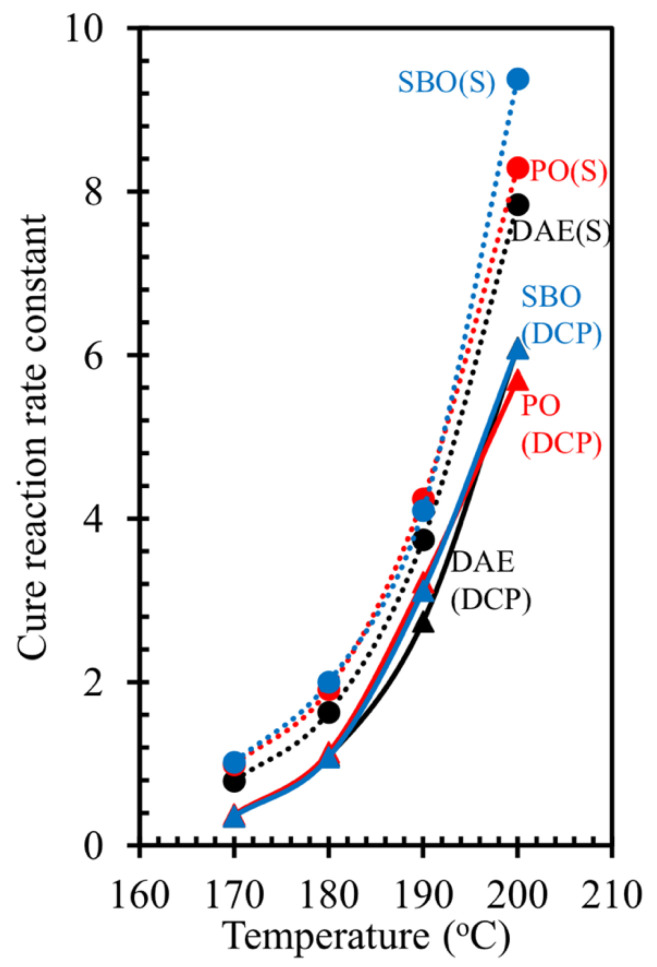
Cure reaction rate constant of NR compounds containing different types of oils under various curing systems as a function of curing temperatures.

The cure kinetics [31], i.e., cure reaction rate constant and activation energy, of the rubber compounds were calculated. Figure 6 shows the typical plot of ln(M_H_ − M_t_) against t of the rubber compounds containing processing oils with different curing systems. The slope of the line for the rubber compounds containing processing oils and cured with sulfur was steeper than that for the rubber compounds containing processing oils and cured using peroxide. Figure 7 shows the cure reaction rate constant of the rubber compounds as determined by the slope of these lines. The different types of processing oils had no influence on the cure behavior of rubber compounds cured with peroxide. Thus, the cure reaction rate constant of these compounds showed no significant change. In addition, results showed that the cure reaction rate constant increases with increasing temperatures. As discussed earlier, the fatty acids in bio-oils can speed up sulfur vulcanization. The cure reaction rate constant of the rubber compounds containing bio-oils and cured with sulfur was higher than that of the compounds containing DAE oil [16].

**Figure 8:**
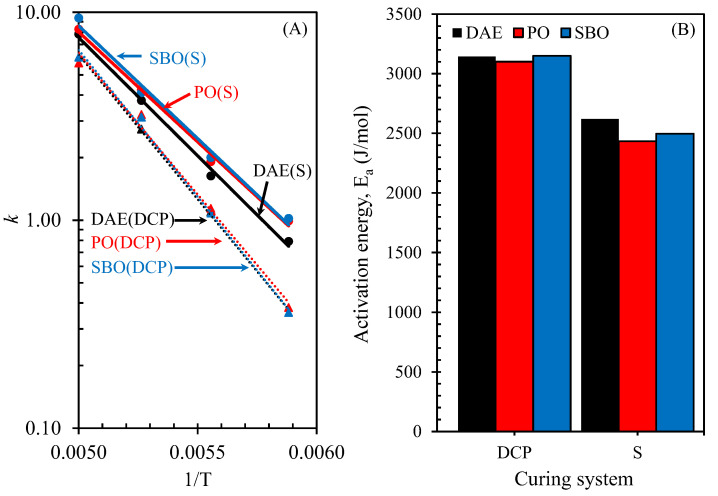
lnk versus 1/T (**A**) and activation energy (**B**) of NR compounds containing different types of oils under various curing systems.

Figure 8A,B present the plot of lnk versus 1/T and the activation energy of the rubber compounds containing processing oils and cured by peroxide and sulfur, respectively. The activation energy was obtained by the slope of the plot of lnk versus 1/T [31]. No significant difference was observed in the activation energies of the rubber compounds containing different types of processing oils and cured with DCP. However, the results showed different behaviors to those of the rubber compounds cured with sulfur. These results revealed that the use of bio-oils in the rubber compounds cured with sulfur decreased the activation energy. This observation supported the proposition that bio-oils accelerate sulfur vulcanization—a short cure time occurs with the increase in cure rate index and cure reaction rate constant [16].

### 3.4. Crosslink Density

**Figure 9:**
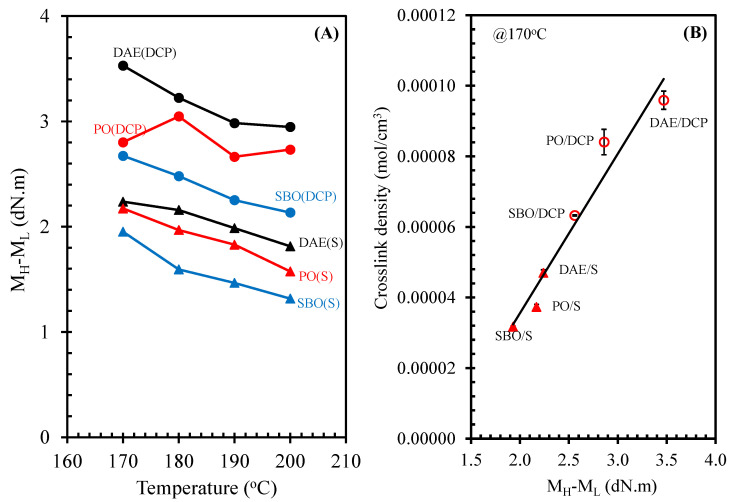
Torque difference (M_H_-M_L_) as a function of curing temperatures (**A**) and the correlation between the crosslink density and the torque difference at a curing temperature of 170 °C (**B**) of NR compounds containing different types of oils under various curing systems.

The degree of crosslinking points within the rubber matrix as a crosslink density is a crucial factor in optimizing end-use properties, such as hardness, modulus, tensile strength, tear strength, and abrasion resistance [1]. As shown in the cure curve of rubber compounds in Figure 4, torque difference, i.e., maximum torque (M_H_)−minimum torque (M_L_), is widely applied to indicate the crosslink density of rubber compounds due to an increase in the torque of unfilled rubber compounds during vulcanization. This increase is affected by the increase in crosslink point within the rubber matrix. Figure 9A shows the torque difference as a function of curing temperatures of NR compounds containing different processing oils and cured by various curing systems. It was found that the bio-oils strongly affected the torque difference of rubber compounds cured by peroxide. The results showed that the torque difference of DCP-cured rubber compounds decreased with the addition of bio-oils. Thus, the use of bio-oils decreases the crosslink density of the DCP-cured rubber compounds. The crosslink density can be measured with equilibrium swelling in toluene and calculated using the Flory–Rehner equation. The crosslink density of the NR compounds containing different types of processing oil were highly correlated with the torque difference obtained from the cure curve (Figure 9B). Among the different types of processing oil, the DAE oil showed a higher crosslink density than the bio-oils in an identical curing system. The addition of bio-oils in the NR compound decreased the crosslink density, in which PO induced a higher level of crosslink density than SBO.

For the peroxide curing system, peroxide radicals are able to abstract a hydrogen atom from polymer chains and any available source, such as antioxidant or processing oil, in the component contained in the rubber compound system. After the peroxide radical abstracts the hydrogen atom from non-polymer chains, such as oil, the crosslink density within the rubber matrix is reduced. Therefore, chemical structure is a main factor influencing the efficiency of abstraction by a radical. The abstraction of allylic hydrogen by a radical has a higher efficiency than that with tertiary, secondary, and primary structures [1]. The structure of bio-oils used in this study, i.e., PO and SBO, consists of three fatty acids attached to a glycerol via an ester linkage. Fatty acids can either be saturated or unsaturated aliphatic hydrocarbons depending on the type. SBO contains more unsaturated fatty acids than PO, as indicated by the ratio of unsaturated and saturated fatty acids. Therefore, the addition of SBO in the NR compounds decreased the crosslink density because of the high efficiency of SBO in the reaction with peroxide curing agent.

**Figure 10:**
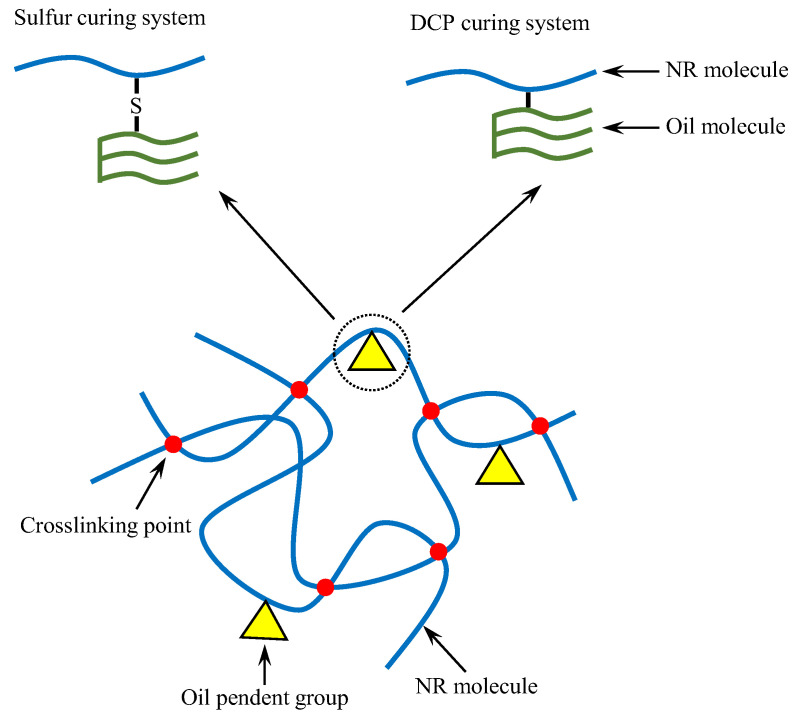
Proposed model for the representation of bio-oils, i.e., PO and SBO, in the rubber compounds cured with different curing systems.

Among the different curing systems, the sulfur-cured rubber compounds showed similar behavior on crosslink density when compared to the ones cured with DCP. In general, the sulfur used as a curing agent for rubber is active toward the carbon double bond (–C=C–) of unsaturated fatty acids [16,32,33]. This property possibly explains the reduction of crosslink density in the sulfur-cured NR compounds with bio-oils. The proposed model for the representation of bio-oils, i.e., PO and SBO, in the rubber compounds cured with different curing systems was shown in Figure 10.

### 3.5. Tensile Properties, Hardness, and Compression Set of NR Compounds

**Figure 11:**
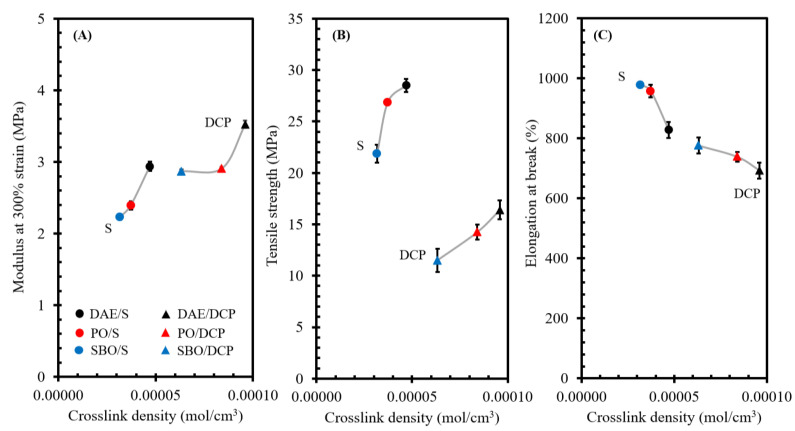
Modulus at 300% strain (**A**), tensile strength (**B**), and elongation at break (**C**) as a function of the crosslink density of NR compounds containing different types of oils and cured by various curing systems.

As discussed in the section of solubility aspects, bio-oils have promoted compatibility with the NR matrix compared with the DAE oil. However, the rubber compounds containing bio-oils did not show superior properties. In this study, the use of different types of oils strongly influenced the crosslink density. Therefore, the correlation of crosslink density and properties of rubber compounds containing different types of oils was further investigated. Mechanical properties, including modulus at 300% strain, tensile strength, and elongation at break, were tested for the rubber compounds containing oils and cured with different curing systems. The results are shown in Figure 11A–C. The rubber compounds containing different types of oils and cured by sulfur showed a lower modulus at 300% strain and higher tensile strength and elongation at break than the rubber compounds cured by DCP. This observation confirmed that the rubber compounds cured with sulfur were more flexible than those cured with DCP. The mechanical properties of rubber compounds containing different types of oils were presented as a function of crosslink density. The modulus at 300% strain and tensile strength of the rubber compounds containing different types of oils cured with DCP increased with the crosslink density, but the elongation at break decreased. These results revealed that the crosslink density within the rubber matrix strongly affected the mechanical properties of rubber compounds. In addition to the crosslink density, the viscosity of the processing oils affected the properties of the rubber compounds, especially elongation at break. It was reported that the use of bio-oils gave higher elongation at break than the ones with DAE. This was due to the low viscosity of bio-oils that promotes a high lubricating effect. So, it could be supported the results in this study that the use of two types of bio-oils, i.e., PO and SBO, in the rubber compounds showed a high elongation at break [34].

As can be seen in Figure 11A–C, the rubber compounds cured with DCP showed the same trend of the mechanical properties with rubber compounds cured with sulfur. This evidence confirmed that the crosslink density within the rubber matrix gave a huge influence on the mechanical properties. As can be seen in Table 2, regardless of curing system, the replacement of DAE with bio-oils produced a similar change in the tensile properties of rubber compounds, including the decreased modulus and tensile strength but increased elongation at break. The change in the modulus of rubber compounds cured with sulfur and peroxide system was nearly similar. However, it was observed that the decrease in tensile strength of rubber compounds cured with sulfur system was less than the ones cured with peroxide. As mentioned, the presence of bio-oils showed a positive effect on elongation at break. It was noted that the increase in elongation at break of rubber compounds cured with sulfur system was higher than the ones cured with peroxide. For rubber formulations used here, it can be inferred that the use of bio-oils is more suitable for sulfur than peroxide curing system.

**Figure 12:**
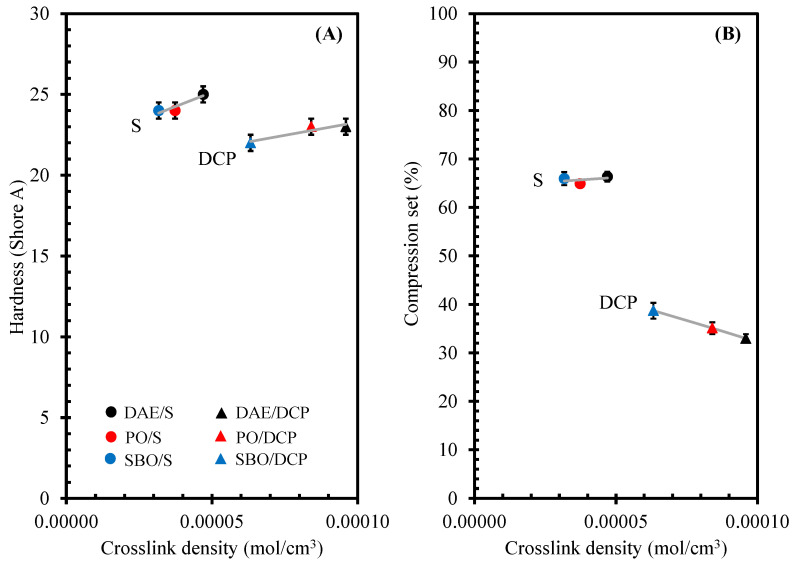
Hardness (**A**) and compression set (**B**) as a function of crosslink density of NR compounds containing different types of oils and cured by various curing systems.

The hardness of rubber compounds containing different types of oils and cured with various curing systems was in Figure 12A. The rubber compounds cured by peroxide curing system exhibited a slightly lower hardness than those cured with sulfur. The hardness of rubber compounds changed when different types of oils were used. As discussed earlier, the addition of bio-oils in the rubber compounds decreased the levels of crosslink density. The rubber compounds containing bio-oils showed a lower hardness than the compounds containing DAE oil under the same curing system. As shown in Figure 12A, the hardness of rubber compounds slightly increased with the crosslink density. In addition, the bio-oils promoted a good lubricating effect due to the low viscosity. Because the low crosslink density and good lubrication effect, the use of bio-oils showed low hardness of the rubber compounds when compared to the ones with DAE.

Compression set is also an important property of rubber compounds and is defined as the amount of permanent deformation of rubbers that occurs when the rubber material is compressed at specific deformation, time, and temperature. Figure 12B shows the compression set of rubber compounds containing different types of oils and cured in various curing systems. The results revealed that the compression set of the rubber compounds was strongly affected by the curing systems. The rubber compounds cured by the peroxide curing system showed a lower compression set than those cured by the sulfur system. This finding was due to the good thermal stability of the –C–C– bond generated by the peroxide curing system, leading to a low compression set. The compression set of rubber compounds containing different types of oils was considered as a function of crosslink density. For the rubber compounds cured with peroxide, the different types of oils induced significant differences in the compression set of NR compounds. The compression set of the rubber compounds decreased with the increase in the crosslink density (Figure 12B). This result was due to the high crosslink density that limited the mobility of rubber chains. Thus, the crosslinked network prevented rubber deformation. However, the compression set did not significantly change when different types of oils were used in the rubber compounds cured by the sulfur curing system.

## 4. Conclusions

The influences of bio-oils, i.e., PO and SBO, on the properties of the unfilled NR compounds cured with peroxide system were investigated here and comparatively discussed with the ones cured with sulfur system from our previous study using DAE-based petroleum oil as a reference. When compared with DAE, the bio-oils did not significantly change the cure time, cure rate index, cure reaction rate constant, and activation energy of the rubber compounds cured by peroxide. However, NR compounds containing bio-oils cured with sulfur showed a shorter cure time, higher cure rate index and cure reaction rate constant, and a lower activation energy than the compounds with DAE. The bio-oils strongly affected the crosslink density of rubber compounds under both curing systems. The bio-oils gave the rubber a lower crosslink density than the DAE oil due to the possible reaction between the curing agents and bio-oil molecules. Considering the lower change in the mechanical properties for the rubber formulations used here, it was proposed here that the replacement of DAE with bio-oils was more applicable for the sulfur cure system than the peroxide one.

## Figures and Tables

**Table 1:** Rubber formulation.

Ingredient	Quantity (phr)
	Peroxide
NR	100
Processing oils *	15
DCP	2

* Three types of processing oils, i.e., DAE, PO, and SBO, were used.

**Table 2:** Tensile properties, i.e., modulus at 300% strain, tensile strength, and elongation at break, of rubber compounds cured with different curing systems and change on the properties of rubber compounds containing bio-oils compared to DAE.

Curing System	Type of Oil	Modulus at 300% Strain		Tensile Strength		Elongation at Break	
		Value (MPa)	Change (%)	Value (MPa)	Change (%)	Value (%)	Change (%)
DCP	DAE	3.53		16.4		690	
	PO	2.91	−18	14.2	−13	740	+7
	SBO	2.87	−19	11.4	−30	780	+11
S	DAE	2.94		28.5		830	
	PO	2.39	−19	26.8	−6	980	+12
	SBO	2.23	−24	21.8	−24	990	+19

## Data Availability

Not applicable.
